# The need for a holistic approach for SSc-ILD – achievements and ambiguity in a devastating disease

**DOI:** 10.1186/s12931-020-01459-0

**Published:** 2020-07-23

**Authors:** Anna-Maria Hoffmann-Vold, Yannick Allanore, Elisabeth Bendstrup, Cosimo Bruni, Oliver Distler, Toby M. Maher, Marlies Wijsenbeek, Michael Kreuter

**Affiliations:** 1 Department of Rheumatology, Oslo University Hospital, Rikshospitalet, Pb 4950 Nydalen, 0424 Oslo, Norway; 2 Institute of Clinical Medicine, University of Oslo, Oslo, Norway; 3 Rheumatology Department, Cochin Hospital, University of PARIS and INSERM U1016, Paris, France; 4 Center for Rare Lung Diseases, Department of Respiratory Diseases and Allergy, Aarhus University Hospital, Aarhus, Denmark; 5 Department of Experimental and Clinical Medicine, Division of Rheumatology, University of Florence, Florence, Italy; 6 Department of Rheumatology, University Hospital Zurich, Zurich, Switzerland; 7 Inflammation, Repair, and Development Section, National Heart and Lung Institute, Imperial College London, London, UK; 8 National Institute for Health Research Respiratory Clinical Research Facility, Royal Brompton Hospital, London, UK; 9 Keck School of Medicine, University of Southern California, 2020 Zonal Avenue, Los Angeles, California USA; 10 Center for Interstitial lung disease and Sarcoidosis, Department of Respiratory Medicine, Erasmus MC, University Medical Center Rotterdam, Rotterdam, The Netherlands; 11 Center for Interstitial and Rare Lung Diseases, Pneumology, Thoraxklinik, Heidelberg University Hospital and German Center for Lung Research, Heidelberg, Germany

**Keywords:** Systemic sclerosis, Interstitial lung disease, Fibrosis, Treatment

## Abstract

Systemic sclerosis (SSc) is a multi-organ autoimmune disease with complex interactions between immune-mediated inflammatory processes and vascular pathology leading to small vessel obliteration, promoting uncontrolled fibrosis of skin and internal organs. Interstitial lung disease (ILD) is a common but highly variable manifestation of SSc and is associated with high morbidity and mortality. Treatment approaches have focused on immunosuppressive therapies, which have shown some efficacy on lung function. Recently, a large phase 3 trial showed that treatment with nintedanib was associated with a reduction in lung function decline. None of the conducted randomized clinical trials have so far shown convincing efficacy on other outcome measures including quality of life determined by patient reported outcomes. Little evidence is available for non-pharmacological treatment and supportive care specifically for SSc-ILD patients, including pulmonary rehabilitation, supplemental oxygen, symptom relief and adequate information. Improved management of SSc-ILD patients based on a holistic approach is necessary to support patients in maintaining as much quality of life as possible throughout the disease course and to improve long-term outcomes.

## Introduction

Systemic sclerosis (SSc) is a multi-organ autoimmune disease with complex interactions between immune-mediated inflammatory processes, uncontrolled fibrosis of skin and internal organs and vascular pathology leading to small vessel obliteration and capillary loss [1]. Interstitial lung disease (ILD) is a common but highly variable manifestation of SSc [2, 3]. It is associated with high morbidity and mortality and has been the leading cause of death related to SSc over decades [2, 4, 5]. Clinically, this heterogeneity results in large inter-patient differences regarding degree and pattern of lung involvement, disease severity, progression rates and clinical outcome, hampering treatment choices in these patients in daily clinical practice [2, 3, 6].

In 2017, the European League Against Rheumatism (EULAR) and the European Scleroderma trials and Research group (EUSTAR) updated their treatment recommendations for SSc (9). From real life data, however, it is known that treatment in daily clinical practise in patients with SSc-ILD vary and differ widely from these recommendations [7–9]. Recently, evidence-based consensus recommendations for the identification and management of ILD in SSc were published to aid clinical guidance on how to identify patients at need for treatment, and which treatment options to use for initiation and escalation therapy [10]. None of the treatment options included in these recommendations have so far shown convincing effects on patient reported outcome measures or symptom scores; however, this may reflect a failure of the available tools. A holistic approach in treatment of ILD in SSc patients is essential to encompass all diverse features associated with this devastating multi-organ disease. Patient care should not only consist of pharmacological but also non-pharmacological therapies as well as other supportive measures [11]. Quality of life (QoL) centred approaches, including symptom relief, should be considered when initiating and/or escalating treatment but have rarely been assessed in patients with SSc-ILD.

In this review, we aimed to elucidate current treatment options and supportive measures for SSc-ILD focusing on evidence-based data and how these treatments effect outcomes and quality of life in SSc patients. We also discuss evidence for non-pharmacological treatment options and other supporting therapies and their impact on QoL and patient reported symptoms. We identify missing evidence and discuss future perspectives on research priorities in SSc-ILD as a joint venture of ILD experts drawn from pulmonology and rheumatology.

## What treatment options are currently recommended and/or used for SSc-ILD patients?

To date, there is no internationally established therapeutic regimen for SSc-ILD but current treatment approaches focus on immunosuppressant therapies [7]. The decision to initiate treatment for SSc-ILD is assessed by the physician and is often based on symptoms, disease severity or disease progression [8, 10, 12]. In 2017, the updated EULAR/EUSTAR treatment recommendations for SSc stated that immunomodulatory therapy should be considered based on an individual risk-benefit evaluation especially in SSc patients with progressive ILD [13]. Dosage and treatment duration should be adapted on an individual basis considering the overall clinical status and response to therapy [13]. The only EULAR/EUSTAR recommended therapy for SSc-ILD was cyclophosphamide, based on the results from the Scleroderma Lung Study I (SLS I) [14] and the FAST trial [15]. Both trials demonstrated an effect of oral cyclophosphamide compared to placebo in SLS I; and intravenous (i.v.) cyclophosphamide followed by azathioprine treatment in FAST. The FAST study showed only a trend favoring cyclophosphamide [15]. In SLS I, there was a statistically significant difference (− 1.0% with cyclophosphamide versus − 2.6%) in forced vital capacity (FVC) after 12 months [14]. However, long term benefits were not maintained after treatment discontinuation [14]. Relevant adverse events were mainly hematologic side effects and pulmonary infections. The EULAR/EUSTAR recommendation did not include the results of the Scleroderma Lung Study II trial (SLS II), as this was published after the publication of the EULAR recommendations [16]. This trial, which was not placebo controlled, compared mycophenolate mofetil (MMF) over 24 months to oral cyclophosphamide given for 12 months, followed by 12 months placebo. The primary endpoint of this study, superiority of 2 years MMF over 1 year cyclophosphamide, was not met, defining it as a negative trial. Notably, the changes in FVC% predicted were similar after 12 and 24 months, showing an improvement in both groups (2.2% versus 2.9% predicted). In addition, there was a numerical imbalance in death rates, mostly due to progressive ILD, favoring MMF (11 deaths in cyclophosphamide; and 5 in MMF). Moreover, MMF was better tolerated than cyclophosphamide, which perhaps might explain fewer premature withdrawals with MMF (32 withdrawals versus 20).

Biological therapeutics have also been assessed as treatment options for SSc-ILD but are facing challenges. For rituximab, a monoclonal anti-CD20 antibody depleting B-cells, randomized controlled trials (RCTs) are lacking. Limited evidence, however, may suggest an association with lung function improvements [17–19]. One study showed a significant improvement in FVC% predicted from 61.3% (SD 11.28) at baseline to 67.5% (SD 13.59) after 24 weeks in the rituximab group suggesting that rituximab is an effective alternative to cyclophosphamide [20]. This trial needs, however, to be interpreted with caution due to the caveats of being an open label, unblinded, single center trial without a placebo group and the choice of the primary outcome (changes from baseline) [20]. The results are also questioned by an observational EULAR study which did not replicate effects of rituximab on FVC changes [21]. Results from the ongoing RECITAL study (NCT01862926) comparing rituximab and cyclophosphamide are pending.

Another monoclonal antibody is tocilizumab, an anti-IL-6R antibody. Evidence for treatment effects on SSc-ILD comes from the phase II FaSScinate trial [22] and the phase III FocuSSed trial comparing tocilizumab with placebo [23]. Both trials included patients with early diffuse cutaneous SSc with recent skin progression and a more inflammatory phenotype, reflected by elevated acute-phase markers [22, 24]. About two-third of the study population had SSc-ILD at baseline. The primary endpoint, delta change in the modified Rodnan skin score (mRSS) was not met in these trials. However, a strong and clinically meaningful effect on FVC% predicted was consistently reported, which was an exploratory endpoint in the phase II and a key secondary endpoint in the phase III trial. The FVC response was, as expected, of greater magnitude in the SSc-ILD subgroup. Other subgroup analyses showed an association with less significant disease progression as defined by FVC decline ≥10% after 48 weeks and suggested an improvement of lung fibrosis in a quantitative lung fibrosis analysis of HRCT [25]. In addition, similar trends were seen in other predefined secondary endpoints. Yet, since the primary objective (improvement of skin fibrosis) of the trials was not met, these data do not fulfill the highest evidence level despite its strong effect size.

Based on similarities in the clinical presentation and outcomes of idiopathic pulmonary fibrosis (IPF) and other fibrosing ILDs including SSc-ILD, the idea emerged that established drugs approved for IPF might also show similar effects in other progressive fibrosing ILDs [26]. An uncontrolled phase II study with pirfenidone in patients with SSc-ILD was conducted and demonstrated feasibility [27]. Effects and safety of the combination of pirfenidone and MMF compared to MMF alone are presently being investigated in the Scleroderma Lung Study III (NCT0322125).

Nintedanib for the treatment of SSc-ILD has been investigated in the Phase III Senscis trial [28]. Here it was shown that nintedanib significantly reduced the rate of FVC decline of patients with SSc-ILD. Based on these results, nintedanib was approved by the Food and Drug association (FDA) in 2019 to reduce lung function decline in patients with SSc-ILD and is now the first approved drug for SSc-ILD in multiple countries worldwide. Absolute treatment effects were smaller, but relative treatment effects were similar to those reported in the INPULSIS trials which assessed nintedanib for the treatment of IPF [29]. Effects of nintedanib differed numerically depending on MMF use, suggesting a potential benefit of combination with MMF on lung function decline. However, randomization was not performed according to MMF use, and patients had to be stable on MMF ≥ 6 months before study inclusion. The side-effect profile with gastrointestinal adverse events, including diarrhoea were more common in nintedanib than in placebo. When baseline SSc associated gastrointestinal symptoms were subtracted, the frequency of these adverse events was similar to the INPULSIS trial. It is however important to address that patients with SSc very frequently have gastrointestinal involvement due to their SSc. Many SSc patients have lower gastrointestinal involvement due to the disease itself, with approximately 30–40% having diarrhoea,bloating and/or small intestinal bacterial overgrowth (SIBO) hampering their QoL substantially [30–32]. It is to date unknown how QoL will be influenced by long-term treatment with nintedanib in regard to potentially, but unknown continued effects on reducing lung function decline on the one side and development of gastrointestinal side effects in addition to their underlying SSc specific gastrointestinal symptoms on the other. SSc patients will need to receive extensive information and to be monitored closely in regard to gastrointestinal involvement and side effects when treated with nintedanib.

In contrast to the Senscis trial, the INBUILD trial included ILD patients with diseases other than IPF with progressive fibrosis, defined by a deterioration in two out of three of the following domains: FVC, high resolution computed tomography (HRCT) and symptoms over the past 24 months prior to study inclusion [33]. The study investigated effects of nintedanib in this progressive fibrosing phenotype including 39 patients with progressive SSc-ILD. The included SSc-ILD patients were marked by a higher frequency of usual interstitial pneumonia (UIP) pattern on HRCT (61.5%) than in the Senscis trial and all were progressive prior to study inclusion. The annual rate of FVC decline was again significantly lower in the nintedanib treated patients compared to placebo, but the trial was not designed or powered to show a benefit of nintedanib in subgroups based on ILD diagnoses including SSc-ILD [33].

Another treatment option discussed in the EULAR/EUSTAR recommendations is autologous hematopoietic stem cell transplantation (HSCT). It is suggested only for selected SSc patients at high risk of organ failure due to significant and rapid disease progression although clear definition of such phenotype is missing [34, 35].. It should also only be performed in experienced centers. HSCT is associated with an improvement in long-term survival and has shown efficacy on FVC changes in SSc compared to cyclophosphamide [35]. However, substantial serious adverse outcomes including early mortality related to procedure have to be considered [34, 35]. Since SSc-ILD patients are at high risk for severe pulmonary infections and potentially respiratory mortality a careful evaluation of risks and benefits on an individual level is required. All potential candidates for HSCT should go through a multidisciplinary evaluation procedure including a structured treatment decision-making process with the patients [36].

The only curative treatment option, similar to other ILDs, might be lung transplantation. However, this treatment possibility is also limited to selected patients. There exist concerns regarding the multi-organ involvement of SSc patients with frequent presence of reflux and esophageal dysfunction resulting in bronchiolitis obliterans syndrome after lung transplantation. Prior to lung transplantation, all patient with SSc should be assessed with esophageal manometry and pH monitoring. In individuals with abnormal reflux, fundoplication has been shown to potentially offer benefit in both survival and reduced rejection following lung transplantation [37, 38]. It has, however, been shown to be associated with severe dysphagia in a subgroup of patients which has a major impact on QoL in these patients [39, 40]. These severe complications need to be considered in the holistic treatment approach of SSc-ILD patients eligible for lung transplantation.

Outcomes of lung transplanted SSc-ILD patients were shown to be comparable to other ILD patients. Of note, there is an ongoing debate whether these results are applicable to a larger SSc population, as patients in these studies were highly selected [41].

**Fig. 1:**
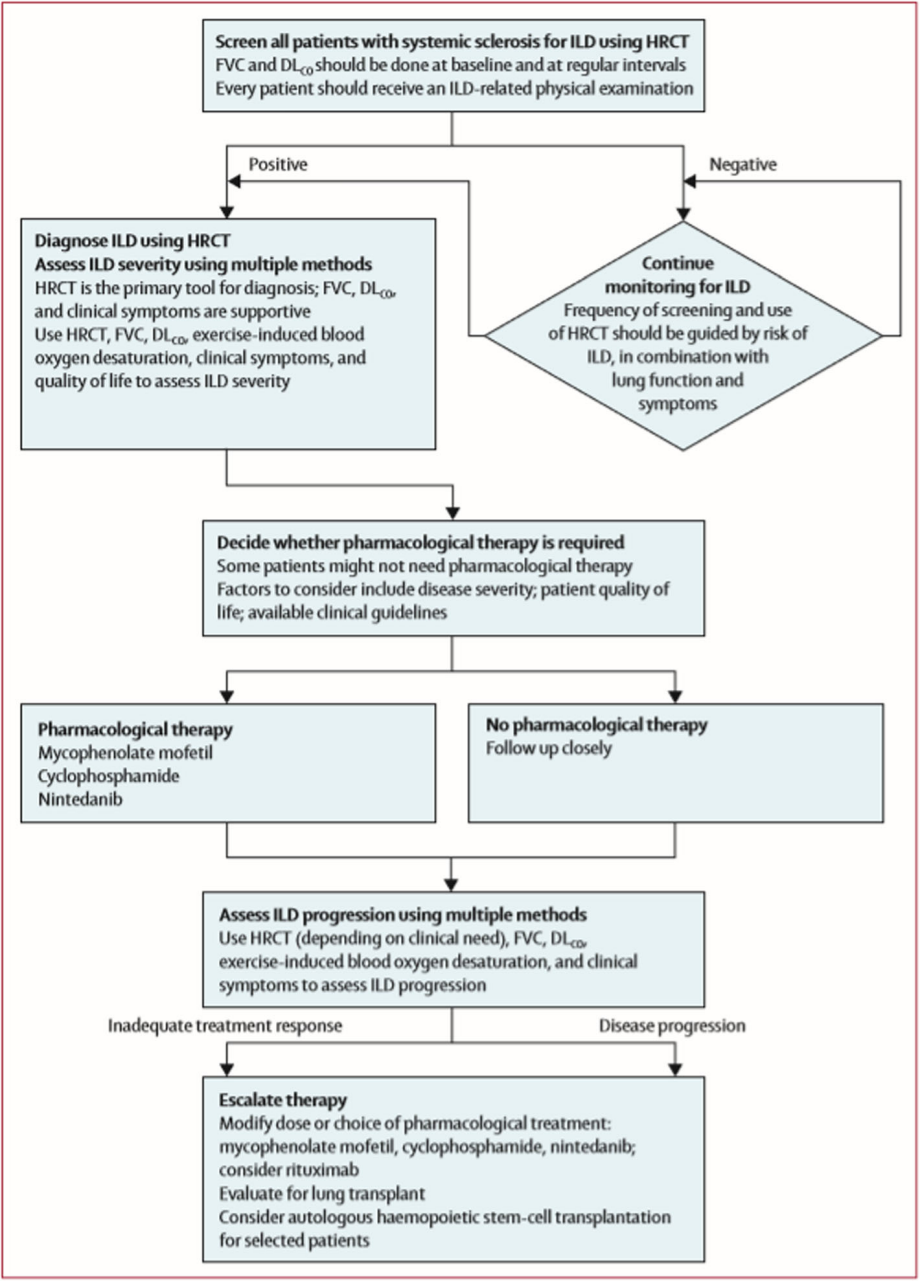
Treatment algorithm for systemic sclerosis associated interstitial lung disease

Very recently, evidence-based consensus statements for the identification and management of SSc-ILD were published. They included statements on treatment initiation, escalation as well as treatment drivers [10]. Except for tocilizumab, all above mentioned treatment options were included (Fig. 1). Cyclophosphamide, MMF and nintedanib were suggested as possible initiation therapies. These options and rituximab despite limited evidence, HSCT and lung transplantation where suggested as escalation treatment options (Fig. 1) [10].

**Fig. 2:**
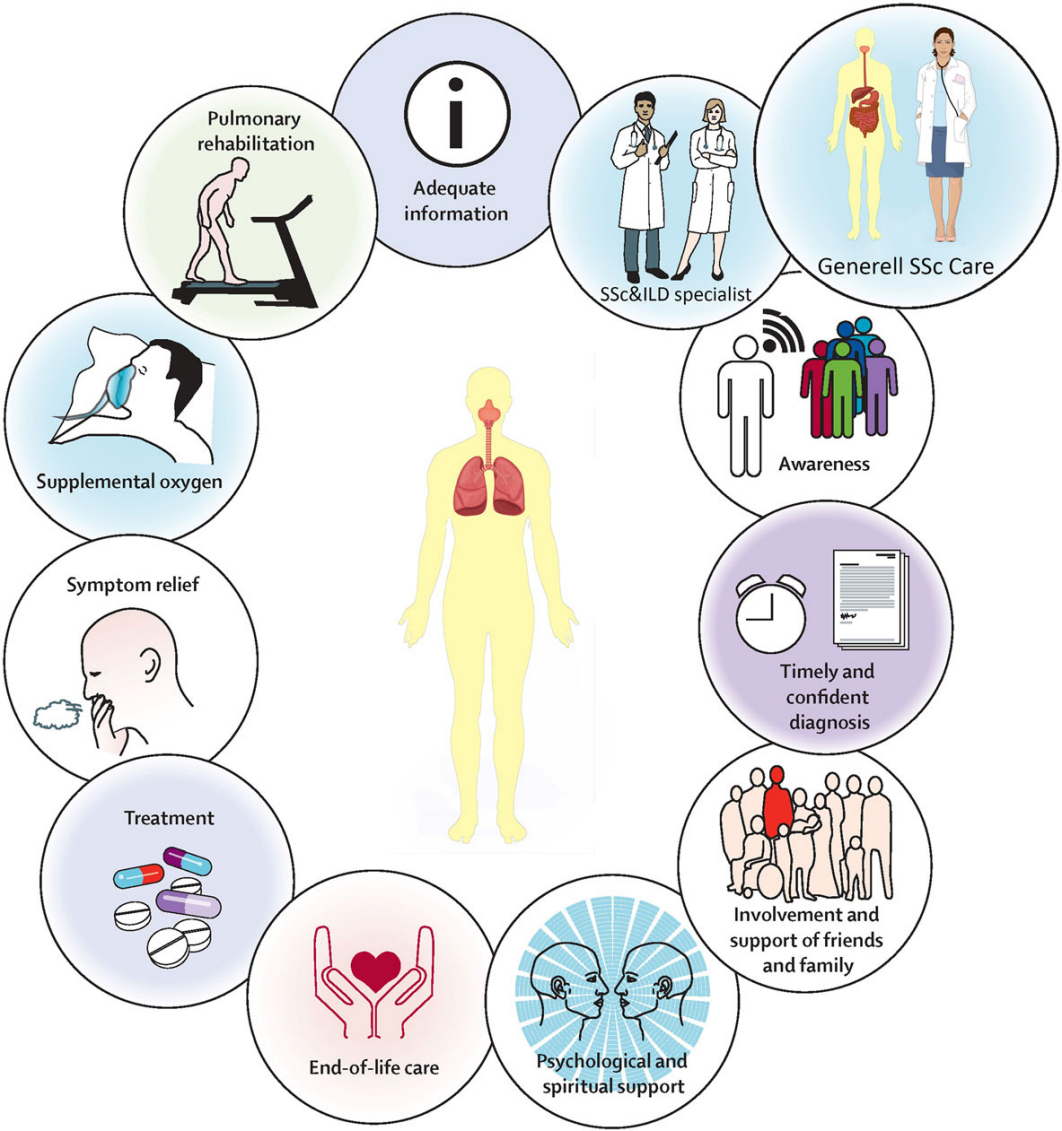
Holistic approach of patients with interstitial lung disease including pharmacological and non-pharmacological treatments and strategies

It is of high importance to emphasize that treatment for ILD in SSc patients strongly depends on the presence of other organ manifestations and the disease course. All SSc patients should therefore be assessed for other organ complications by SSc experts and the choice of specific therapies should be based on a holistic approach (Fig. 2).

## What drives the choice of drugs for patients in first line and subsequent lines, and is supported by the existing literature?

As there are several treatment options currently available for SSc-ILD, it is important to identify patients who are more likely to benefit from a specific drug with an acceptable toxicity profile. To date, available evidence is acquired by using these drugs as monotherapy. Another treatment concept, however, is combination regimens with either upfront combination or a sequential add-on of drugs. It is unknown whether these regimens will provide better outcomes with an acceptable toxicity profile. Additionally, we identified the following lack of evidence regarding the choices of drugs: how patient characteristics and patient preferences affect the choice of a drug; when to switch from one to another treatment and when to escalate treatment defined as either an increase in dose of existing therapy or the addition of a new therapy; what should be the appropriate duration of treatment and finally, how to define treatment success and/or failure in the individual patients.

All SSc-ILD trials showing treatment efficacy involved SSc patients with established ILD at the time of inclusion [14, 16, 28]. In these studies, mean disease duration was about 3 years, 53–63% had diffuse cutaneous SSc and mean baseline FVC was 67–72%, mirroring the general SSc-ILD population seen in daily clinical practice. In comparison, SSc patients included in the tocilizumab trials were selected for worsening skin disease and increased acute phase reactants regardless of the presence of ILD, but with a majority of patients having early and mild ILD [22]. The treatment effect was largely driven by patients with evidence of ILD at baseline, which suggests that tocilizumab might reduce progressive disease in early, active stages with ongoing inflammation in diffuse cutaneous SSc ILD patients and preserved lung function. However, it is of importance to note that the various trials had different study designs and sizes. Of even more importance, the patient cohorts substantially differed between these studies in their clinical SSc profile, e.g. the SSc-ILD trials were not enriched for early, inflammatory diffuse cutaneous SSc patients. This makes a direct comparison of the efficacy between the different studies unreliable.

## Does current treatment improve outcomes in patients with SSc-ILD?

In order to truly evaluate the benefits of pharmacologic as well as non-pharmacologic treatments, reliable and valid outcome measures are needed, but very few have been validated in SSc-ILD. OMERACT (Outcome Measures in Rheumatology), a group of independent international health professionals and patient research partners, have strived to systematically identify and validate outcome measures in SSc and other connective tissue diseases (CTDs) based on the concepts of truth, discrimination and feasibility [42]. To answer whether current treatment meaningfully improves outcome in patients with SSc-ILD we evaluated all conducted RCT trials in SSc with the included outcome measures (Table 1).

**Table 1:** Phase I, II and III trials in systemic sclerosis with lung assessed as a primary or secondary endpoint

Publication	No. patients included	Treatment	Primary endpoint	Secondary endpoint
Distler et al. NEJM 2019 [28]	576	Phase III Nintedanib 150 mg × 2	**Annual rate of Decline in FVC**	Absolute changes in: • mRSS • SGRQ at week 52 • **FVC (ml)** • %DLCO • Net digital ulcer burden • HAQ-DI • FACIT–Dyspnea questionnaire **Annual rate of decline in %FVC** Time to death from any cause
Sircar et al. Rheumatology 2018 [20]	64	Phase II Monthly pulses of CYC 500 mg/m2 or RTX 1000 mg × 2 doses at 0, 15 days	**%FVC at 6 months**	Absolute change in: • **FVC (l)** • mRSS^a^ • **6MWTD** • **Medsgers score**^a^ • New onset/ worsening of pulmonary hypertension
Hsu et al. J Rheumatol 2018 [43]	23	Phase II Pomalidomide 1 mg q.d.	FVC Total UCLA SCTC GIT V2.0 score mRSS	BDI/TDI Pulse oximetry (SpO2) UCLA SCTC GIT 2.0 subscale scores SHAQ
Khanna et al. Ann Rheum. Dis. 2017 [22]	87	Phase II Tocilizumab 162 mg sc	mRSS	%FVC %DLCO VAS (Clinician Global) HAQ-DI, Patient Global VAS FACIT-Fatigue Score Pruritus 5-D Itch Scale.
Tashkin et al. LRM 2016 [16]	142	Phase II Oral CYC 2 mg/kg/day or MMF 1500 mg b.i.d.	%FVC at 24 months	%DLCO TDI mRSS LCQ Change in HRCT extent
Khanna et al. J Rheumatol 2016 [27]	63	Phase II Pirfenidone 801 mg t.i.d.	Safety	UCLA SCTC GIT V2.0 score FVC DLCO Mahlers dyspnea score TDI HAQ-DI PtGA mRSS
Burt et al. Lancet Resp. Med. 2011 [44]	19	Phase II HSCT vs. monthly pulses of IV CYC 1 g/m^2^	mRSS, 25% decrease FVC, 10% improvement	CT volume of lung disease DLCO TLC
Spiera et al. Ann Rheum Dis 2011 [45]	30	Phase IIa Imatinib 400 mg q.d.	mRSS	FVC DLCO SF36 SHAQ-DI VAS (global, SOB, pain, Raynaud)
Seibold et al. Arthrit. Rheum. 2010 [46]	163	Phase II Bosentan 125 (62.5 mg) mg b.i.d.	6MWTD	Death FVC DLCO BDI mRSS Medsgers score SHAQ-DI VAS
Denton et al. Arthrit. Rheum. 2007 [47]	45	Phase I/II Recombinant Human Anti–Transforming Growth Factor Antibody Therapy (CAT-192)	mRSS	FVC TLC DLCO HAQ VAS (global, Raynaud, lung disease, GO disease, digital ulcers)
Tashkin et al. NEJM 2006 [14]	158	Phase II Oral CYC 1–2 mg/kg q.d.	FVC	DLCO TLC HAQ SF36 Mahler dyspnea score VAS breathing
Hoyles et al. Arthrit. Rheum. 2006 [15]	45	Phase II 20 mg oral prednisolone (alternate days), 6 monthly IV pulses of CYC 600 mg/m^2^ followed by AZA 2.5 mg/kg/day	FVC DLCO	Dyspnea score Change in HRCT extent and pattern
Nadashkevich et al. Clin Rheumatol. 2006 [48]	60	Phase I/II Oral CYC 2 mg/kg for 12 months, then 1 mg/kg for 12 months vs. Aza 2.5 mg/kg for 12 months, then 2 mg/kg for 18 months		**mRSS**^**b**^ **Raynaud frequency** %FVC %DLCO
Binks et al. Ann. Rheum. Dis. 2001 [49]	41	Phase I/II HSCT	Mortality Disease progression	mRSS VC DLCO LVEF by echocardiography

Bold: Significant change; ^a^ CYC improved only mRSS and Medsgers score; RTX improved all secondary outcomes. ^b^Improvement with CYC, no improvement with Aza

*FVC* Forced vital capacity, *DLCO* Diffusing capacity for carbon monoxide, *TLC* Total lung capacity, *VC* vital capacity, *mRSS* modified Rodnan skin score, *6MWTD* 6 min walk test distance, *SGRQ* St. Georges respiratory Questionnaire, *HAQ-DI* Health Assessment Questionnaire Disability Index, *FACIT–Dyspnea* Functional Assessment of Chronic Illness Therapy – Dyspnea, *UCLA SCTC GIT* University of California, Los Angeles Scleroderma Clinical Trial Consortium Gastrointestinal Tract, *BDI/TDI* Baseline and transition dyspnea index, *LCQ* Leicester Cough Questionnaire, *SHAQ* Scleroderma Health Assessment Questionnaire, *PtGA* Patients global assessment of disease activity, *CYC* cyclophosphamide, *MMF* mycophenolate mofetil, *HSCT* hematopoic stamcell transplantation, *LVEF* left ventricular ejection fraction, *VA* Visual Analogic Scale

Lung physiology has been the preferred outcome measure so far. Pulmonary function tests with FVC, total lung capacity (TLC), and diffusing lung capacity for carbon monoxide (DLCO) have frequently been used in phase II and III RCTs in SSc (Table 1). FVC is the most widely used variable to reflect the level of restrictive lung function impairment [50]. It is a validated outcome measure for lung disease in SSc and was used as the primary endpoint in three recent trials [14, 20, 28]. FVC measured as annual rate of decline and as an absolute value was able to differentiate between treatment arms in both SLS I and SENSCIS. TLC is also a measure for restriction while DLCO is sensitive for lung parenchymal changes but not specific as it also measures changes due to vasculopathy, emphysema or anemia. Both parameters have been used as secondary outcome measures in several trials without being able to show any significant improvement although trends were found [14–16, 22, 27, 28, 44–49] (Table 1).

The extent of ILD assessed by HRCT has been used as secondary outcome in few studies. HSCT showed significantly reduced ILD extent in one study [44], whereas treatment with cyclophosphamide and MMF favored but did not show any significant changes in two studies [15, 16]. It is likely that quantitative assessment of HRCT images by lung texture analysis as well as artificial intelligence (AI) may be useful in future studies as these methods not only identify and quantify ILD patterns (i.e. ground-glass, reticular patterns, honeycombing) but can also asses vascular involvement [51, 52].

The 6-min walk test as a functional assessment was used as the primary endpoint parameter in a trial investigating bosentan for SSc-ILD and as a secondary parameter comparing cyclophosphamide with rituximab and was found useful [20, 46]. However, the 6-min walk test lacked correlation with standard physiologic parameters for ILD probably because it also can reflect other SSc manifestations such as vascular and musculoskeletal involvement and pain [53, 54].

Hospitalization, exacerbations and mortality have often been used as outcome measures in ILD trials but so far not as a primary outcome measure in any prospective SSc-ILD trial. Time to death or death were included as secondary outcomes in two trials without reaching statistical significance most likely due to the relative short study duration of 1–2 years [28, 46].

There have been identified a number of circulating biomarkers for SSc-ILD including Krebs von den Lunge 6 (KL-6), surfactant protein D (SP-D) and serum chemokine (C-C motif) ligand 18 (CCL18), but none of them are fully validated and have not been used as outcome measures in SSc-ILD trials [55, 56].

## Does current treatment improve quality of life in patients with SSc-ILD?

Quality of life assessment is increasingly requested by patients and by health authorities in research and as outcome parameters in RCTs. Despite increasing data about treatment effects on functional and clinical outcomes, data on QoL, disability and physical/mental function has traditionally been less well assessed. When considered in their totality, the results have not been very encouraging. QoL is commonly captured by patient related outcome measures (PROMs) which are based on reports directly from the patient and describe patients’ perception of their own health status or QoL. They may diverge from physicians’ needs and interests and often measure different perspectives than those captured by physiologic measures [57, 58]. No PROM specific for SSc-ILD exists. The most widely used lung specific QoL score is St. George’s Respiratory Questionnaire (SGRQ), originally developed for obstructive lung disease but also validated in restrictive lung diseases according to the OMERACT [59, 60]. Also, the health assessment questionnaire disability index (HAQ-DI) developed for rheumatoid arthritis, functional assessment of chronic illness therapy (FACIT)-fatigue and FACIT-dyspnea are commonly used. Other PROMs are Mahler’s dyspnea score, baseline and transition dyspnea index (BDI/TDI), Leicester cough score and Visual Analogic Scale (VAS) scores and the Kings Brief Interstitial Lung Disease Questionnaire (KBILD). Short Form 36 questionnaire (SF36), a generic QoL score, represents the most frequently tested functional measurement [61]. None of these scores and questionnaires have so far shown statistical difference in SSc-ILD trials (Table 1). Since none of the discussed trials reached more than the primary outcome (only Senscis) formal analysis of more secondary endpoints including all PROs were not allowed.

Despite not reaching statistically significance, changes in some of the domains of these questionnaires could still represent clinically meaningful variation in patient’s perception and were therefore included in our review.

HAQ-DI is the most commonly used disability index in RCTs, with a reported minimally clinical important difference (MCID) of 0.10–0.20 [62]. In SLS 1, cyclophosphamide determined MCID decline for HAQ-DI at 12 months (improvement), with significant differences compared to placebo lasting up to 18 months. Despite treatment interruption, some domains of SF-36 also showed statistically differences with respect to the placebo treated group [14]. Impact on QoL was replicated in two HSCT trials for diffuse cutaneous SSc [34, 35]. In these RCTs, patients on cyclophosphamide showed a decline of HAQ-DI (improvement) of more than the MCID, which was even more pronounced in the transplantation arm [34, 35]. Similarly, SF-36 improved significantly, with superiority of transplant over cyclophosphamide for the physical component only [34].

MMF, despite its widespread use, has little evidence available to support an impact on QoL [14, 63].

Tocilizumab, when tested in diffuse cutaneous SSc in the phase II FaSScinate trial, showed improvement in both HAQ-DI and FACIT with MCID reached, as well as in clinician and patient global VAS [22].

Rituximab, in addition to background therapy regardless indication (not specifically SSc-ILD), determined a MCID decrease of HAQ-DI in a small RCT, which did not reach significance, comparing 1 year with baseline data [17]. Despite a different patient target, these data were partially confirmed by the EUSTAR cohort data, in which a decrease of HAQ-DI was seen between baseline and last follow-up when rituximab was used for musculoskeletal involvement [21]. A limitation with this analysis is, however, that spontaneous regression of the mRSS is included, which is a driver of HAQ-DI in diffuse cutaneous SSc.

Regarding anti-fibrotic therapy, despite beneficial effects in arresting lung function decline by nintedanib, no significant improvement in any PROM has been reported so far. In the SENSCIS study, both change in SGRQ, HAQ-DI and FACIT-dyspnoea questionnaire at 52 weeks were assessed. Nintedanib did not reach MCID changes in HAQ-DI. Similarly, despite no MCID being available for SSc patients, the FACIT-Dyspnoea questionnaire and the SGRQ showed no significant change compared to placebo [28].

## What is the evidence for other supporting measures, palliative care and their impact on quality of life?

Traditionally, trials on the treatment of ILD have focused on pharmacological therapies and little attention has been paid to non-pharmacological treatment, supporting therapies and holistic approaches (Fig. 2). Patients with SSc-ILD, as do most other patients with ILD, face many challenges in coping with their disease [64]. Besides practical issues these include; lack of information and difficulties in access to specialist care, symptom burden, disease progression, the negative impacts of stress and depression [64–66]. We identified little evidence-based data on non-pharmacological treatment and supportive care specifically for SSc-ILD, but some studies in ILD or in SSc have also included small numbers of patients with SSc-ILD.

Pulmonary rehabilitation is often recommended for SSc-ILD, but the data to support this recommendation is scarce. A recent systematic review on SSc overall population and pulmonary rehabilitation concluded that exercise therapy is considered safe, but that no definite conclusion on its efficacy can be drawn [67]. An RCT including patients with ILD also recruited a subgroup of 19 patients with CTD-ILD. Whilst the trial showed clinical meaningful effects on 6-min walk distance and QoL in the overall populations, the effect was limited in the CTD-ILD group [68]. Although the beneficial effect of pulmonary rehabilitation in ILD is acknowledged, more disease specific studies are needed to prove its beneficial effect also for SSc-ILD [69, 70]. It may well be that commonly found extrapulmonary manifestation of SSc, such as skin, joint and muscle involvement, demand other or additional training approaches.

No specific guidance on the use of supplemental oxygen in SSc-ILD exists. In patients with ILD, supplemental oxygen is recommended if severe hypoxemia at rest is present, though there is no structured research to support these recommendations [71]. In daily clinical practice, in most patient with ILD and hypoxia supplemental oxygen is started, but there is a large variability in approaches [72]. In patients with ILD of different etiologies and hypoxia on exertion, a first prospective RCT with ambulatory oxygen showed a positive effect on QoL and dyspnea [73]. Ten percent of the patients in this study had a diagnosis of CTD-ILD. This study supported offering supplemental oxygen to patients with an oxygen desaturation measured with pulse-oximetry of ≤88% on a six minutes walking test.

Palliative care comprises symptom management, supportive measures and end-of-life care; and aims to improve QoL throughout the entire disease course and a dignified death. To date, data on palliative care in SSc-ILD is lacking. Despite the paucity of studies on palliative measures in SSc-ILD, data from ILD and the respiratory field may give some guidance [73, 74]. Symptoms often encountered in SSc-ILD are, similar to other patients with fibrosing ILDs, cough, dyspnea, fatigue and depression.

For the relief of dyspnea often a combination of non-pharmacological and pharmacological measures may be needed. A longitudinal cohort study in patient with fibrosing ILD starting on long-term oxygen showed that opioids and low dose benzodiazepines could safely be used [74]. The results of a RCT on the effect of opioids for fibrosing ILD are pending (NCT02622022). A positive effect on breathlessness mastery and survival was found in an RCT of a breathlessness support service, which also included 19 patients with ILD [75].

Cough is often a refractory symptom in fibrotic ILD and no evidence-based treatment recommendations exist to date. In the SLS II study, treatment with MMF or oral cyclophosphamide was associated with a decrease in reported cough frequency, but no effect on cough related QoL was found [76].

Causes of fatigue and depression in ILD and SSc-ILD are often multifactorial [64, 65, 77]. A comprehensive and structured approach is recommended both for ILD-related symptoms as well as extrapulmonary symptoms and co-morbidities [11, 77]. As the prognosis of progressive fibrosing SSc-ILD may be poor, advanced care planning and end-of-life care should also be part of a comprehensive supportive treatment approach. In fibrosing ILD multidisciplinary care programs including palliative care specialists resulted in better symptom control, improved QoL and more people dying at home, i.e. respecting patients´ and caregivers´ desire for a respectful death amongst the beloved ones [78, 79].

Besides supportive measures, preventive advice is given in line with recommendations to all ILD patients, mostly based on expert opinion and common sense. In the case of smoking, vaping or other substance abuse, patients should strongly be encouraged to stop. Influenza and pneumococcal vaccinations are offered to all patients to prevent infections that may trigger an exacerbation or worsening of disease.

## Future perspectives on research priorities in SSc-ILD

Current clinical practice, supported by treatment recommendations as discussed above, is to screen all SSc patients for ILD and initiate treatment of patients with SSc-ILD if apparent disease, defined by the presence of severe ILD on HRCT, restrictive lung disease or declining lung function. This clinical practice is partly influenced by the incomplete understanding of which patients are at risk of early progressive ILD, and the knowledge that only about 30% of SSc-ILD patients are progressive in the following year (65, 66). It is however known that lower lung function, higher extent of ILD on HRCT and declining lung function, as well as other SSc specific risk factors as diffuse cutaneous SSc and anti-topoisomerase I antibodies, both associated with the presence of a severe ILD, is associated with a high mortality. On this background, it is apparent that early identification of patients at risk of progression, before loss of lung function and irreversible lung damage, is of high importance to initiate targeted treatment early and improve clinical outcome. In daily clinical practice SSc-ILD patients usually receive immunosuppressants. Nintedanib, the first targeted SSc-ILD treatment has been approved in many but not all countries already. To date, however, there is a lack of robust evidence to support the long-term efficacy of treatments or to guide their use. It seems tempting that novel treatment concepts should aim for prevention of progression to avoid irreversible organ damage. Further RCTs in different patient populations and head to head comparisons of the currently used drugs are needed to assess the treatment efficacies of available therapeutics for SSc-ILD patients. More research and RCTs on upfront combination and/or sequential use of immunosuppressive and nintedanib will hopefully be conducted in the future. Other targeted therapies that inhibit key pathways in the pathogenesis of SSc-ILD are also needed to expand the ILD treatment armamentarium and further improve patient outcomes.

Many of the previously conducted RCTs in SSc have failed their primary outcome. The frequently used mRSS failed in recent clinical trials as a surrogate parameter for universal disease progression. Other clinical trial concepts i.e. time to worsening using worsening of organ involvement as a study endpoint with trial cohort enrichment for SSc patients at risk for overall disease worsening have been recently suggested and seem promising [80].

Lastly, structured research on supportive measures and on palliative care in SSc-ILD is extremely limited to date and therefore much needed. Despite advances in the treatment SSc-ILD, no clear positive effect on QoL has been shown and many patients still progress and eventually die of their disease. Better measures are needed to support patients in maintaining as much QoL as possible throughout the disease course and thus holistic approaches will need more focus in the years to come.

## Data Availability

Not applicable.

## Abbreviations

BDI/TDI: Baseline and transition dyspnea index
DL_CO_: Diffusing capacity of the lung for carbon monoxide
EULAR: European League Against Rheumatism
EUSTAR: European Scleroderma trials and Research group
FACIT: Functional assessment of chronic illness therapy
FVC: Forced vital capacity
HAQ-DI: Health assessment questionnaire disability index
HRCT: High-resolution computed tomography
HSCT: Haematopoic stemcell transplantation
KBILD: Kings Brief Interstitial Lung Disease Questionnaire
ILD: Interstitial lung disease
MCID: Minimal clinical important difference
MMF: Mycophenolate mofetil
mRSS: Modified Rodnan skin score
PROM: Patient reported outcome measure
RCT: Randomized clinical trial
SF-36: Short Form 36 questionnaire
SGRQ: St. George’s Respiratory Questionnaire
SLS: Scleroderma lung study
SSc: Systemic sclerosis
SSc-ILD: Systemic sclerosis-associated interstitial lung disease
TLC: Total lung capacity
VAS: Leicester cough score and Visual Analogic Scale
QoL: Quality of life
